# Estimation of allele-specific *Ace*-*1* duplication in insecticide-resistant *Anopheles* mosquitoes from West Africa

**DOI:** 10.1186/s12936-015-1026-3

**Published:** 2015-12-18

**Authors:** Luc S. Djogbénou, Benoît Assogba, John Essandoh, Edi A. V. Constant, Michel Makoutodé, Martin Akogbéto, Martin J. Donnelly, David Weetman

**Affiliations:** Institut Regional de Santé Publique de Ouidah/Université d’Abomey-Calavi, Cotonou, Benin; Department of Vector Biology, Liverpool School of Tropical Medicine, Pembroke Place, Liverpool, UK; Centre de Recherche Entomologique de Cotonou, Cotonou, Benin

## Abstract

**Background:**

Identification of variation in *Ace*-*1* copy number and G119S mutation genotype from samples of *Anopheles gambiae* and *Anopheles coluzzii* across West Africa are important diagnostics of carbamate and organophosphate resistance at population and individual levels. The most widespread and economical method, PCR–RFLP, suffers from an inability to discriminate true heterozygotes from heterozygotes with duplication.

**Methods:**

In addition to PCR–RFLP, in this study three different molecular techniques were applied on the same mosquito specimens: TaqMan qPCR, qRTPCR and ddPCR. To group heterozygous individuals recorded from the PCR–RFLP analysis into different assumptive genotypes K-means clustering was applied on the Z-scores of data obtained from both the TaqMan and ddPCR methods. The qRTPCR analysis was used for absolute quantification of copy number variation.

**Results:**

The results indicate that most heterozygotes are duplicated and that G119S mutation must now be regarded as a complex genotype ranging from primarily single-copy susceptible Glycine homozygotes to balanced and imbalanced heterozygotes, and multiply-amplified resistant Serine allele homozygotes. Whilst qRTPCR-based gene copy analysis suffers from some imprecision, it clearly illustrates differences in copy number among genotype groups identified by TaqMan or ddPCR. Based on TaqMan method properties, and by coupling TaqMan and ddPCR methods simultaneously on the same type of mosquito specimens, it demonstrated that the TaqMan genotype assays associated with the K-means clustering algorithm could provide a useful semi-quantitative estimate method to investigate the level of allele-specific duplication in mosquito populations.

**Conclusions:**

*Ace*-*1* gene duplication is evidently far more complex in *An. gambiae* and *An. coluzzii* than the better-studied mosquito *Culex quinquefasciatus*, which consequently can no longer be considered an appropriate model for prediction of phenotypic consequences. These require urgent further evaluation in *Anopheles*. To maintain the sustained effectiveness carbamates and organophosphates as alternative products to pyrethroids for malaria vector control, monitoring of duplicated resistant alleles in natural populations is essential to guide the rational use of these insecticides.

**Electronic supplementary material:**

The online version of this article (doi:10.1186/s12936-015-1026-3) contains supplementary material, which is available to authorized users.

## Background

Copy number variations (CNVs) are defined as DNA sequences ranging from 1 kb to few Mb that have different numbers of repeats within or among individuals [1] and arise from the duplication or deletion of DNA segments [2]. In the human genome, CNVs have been shown to be associated with several phenotypic effects [3–5]. Gene duplication is also thought to be the main potential source of material for the evolution of new gene functions [6] providing an important source of adaptive variation [7]. Several models have been proposed for the evolution of new functions through duplication, most based on ancient events [8], but duplication may also be important for adaptation to changing environmental conditions on a contemporary timescale.

Acetylcholinesterase (AChE) is the target of organophosphate and carbamate insecticides and catalyzes the hydrolysis of the neurotransmitter acetylcholine to terminate nerve impulses at the postsynaptic membrane. Mutations in the *Ace*-*1* gene, which codes for AChE in insects such as the primary African malaria vector *Anopheles gambiae* species pair (*Anopheles gambiae s.s*. and *Anopheles coluzzii*), can confer resistance to carbamate and organophosphate compounds [9]. Of the mutations in the coding sequence of the *Ace*-*1* gene recorded to date, only one, G119S (a single amino acid substitution, from a glycine to a serine at the position 119 in the AChE catalytic site using *Torpedo californica* nomenclature), is found in *Anopheles*, and causes strong resistance to both organophosphates and carbamates [10, 11]. This substitution to the resistant allele (*Ace*-*1*^*R*^) has a high fitness costs in insecticide-free environments [12, 13]. In *Culex pipiens*, *Ace*-*1*^*R*^ has a very similar resistance: fitness cost profile [12]. However, duplicated alleles have evolved on several occasions in *Culex* which link a resistant allele (*Ace*-*1*^*R*^) with a susceptible allele (*Ace*-*1*^*S*^) on the same chromosome in permanent ‘heterozygosity’ [14] alleviating significant costs in field populations [15].

Subsequent discovery of an *Ace*-*1* gene duplication event in *An. gambiae* [16] was thus a major concern for vector control. Furthermore, absence of sequence diversity in duplicated alleles argued for a single origin, despite detection in both *An. gambiaes.s.* and *An. coluzzii* from Burkina Faso and Côte d’Ivoire [16], and Ghana where it was found only on *An. gambiaes.s*. [11], suggesting that spread had already occurred.

An important goal is the development of methods capable of discovering *Ace*-*1* gene copy number variations in field samples of mosquitoes in addition to genotyping G119S mutation alleles. Very recently, a qRT-PCR method to detect duplication was applied to individual female *An. coluzzii* from a multi-insecticide resistant population from Tiassalé, Côte d’Ivoire [17]. In Tiassalé most females are heterozygous at the *Ace*-*1* locus [10, 18]. Heterozygous individuals surviving bendiocarb exposure exhibited both a significantly higher resistant/susceptible allele ratio (*Ace*-*1*^*R*^/*Ace*-*1*^*S*^) in the standard TaqMan genotyping assay and a higher *Ace*-*1* gene copy number, assessed by the qRT-PCR, i.e. they survived because they possessed more copies of resistant alleles [10]. This first demonstration of a direct impact of *Ace*-*1* gene copy number variation on insecticide resistance suggested that *An. gambiae* exhibits greater complexity of *Ace*-*1* gene amplification (cf. ‘duplication’) than previously suspected, with potentially many gene copies and multiple resistant alleles. Moreover the results confirmed fears that *Ace*-*1* copy number variation represents an emergent threat to vector control [19].

To investigate this effectively, and link *Ace*-*1* gene copy number variation to insecticide resistance and fitness costs more widely in the field, widely-applicable detection methods for duplicated alleles are required. Understanding the distribution and spread of the frequency of the *Ace*-*1*^*R*^ allele, particularly when coupled with duplication is of major concern for vector control programmes using carbamates and organophosphates for indoor residual spraying (IRS).

No simple test is available to detect and study *Ace*-*1* duplication in mosquito species due to the lack of sequence features specific to copied alleles. Traditional PCR that visualizes PCR products run on a gel cannot readily discriminate duplicated alleles, which typically display as classical heterozygotes. Djogbenou et al. [19] attempted to estimate the duplicated allele (*Ag*-*Ace*-*1*^*D*^) frequency in a field population by using an indirect method previously developed for *Cx. pipiens*, but such statistical methods may conflate overdominance with duplication [20]. Approaches have been developed to screen for CNVs systematically at a whole-genome level in whole genome sequencing data [21, 22]. However these methods cannot be applied easily to field populations, especially in resource-limited West African settings wherein the *Ace*-*1* gene duplication is found in major malaria vector populations. Due to the lack of validated, field-applicable diagnostic tools, key questions arising from the previous research remain open:

- How frequently does *Ace*-*1* gene duplication occur in field populations?
- What is the extent and consistency of duplication of susceptible and resistant alleles?
- What is the contribution of duplicated alleles to insecticide resistance and other phenotypic traits in the field?

Providing responses to the above questions will help to better evaluate the potential consequences of the *Ace*-*1* gene duplication event for *An. gambiae* resistant population management and on malaria control.

In this study, PCR–RFLP and TaqMan genotyping assays, qRT-PCR to detect copy number, and a newer digital droplet PCR method were applied to the same *An. gambiae* samples to explore variation in *Ace*-*1* gene copy number across West Africa with the aim to identify appropriate strategies for identifying variation at population and individual levels in the main malaria vector.

## Methods

### Mosquito samples and DNA extraction

The samples used in this study were field-collected adults originating from various locations across four West African countries (Table 1) and laboratory strains of known G119S genotype. Some of the field-collected samples were identified for inclusion by implementation of a duplicated haplotype detection protocol based on laboratory crossing and resistance phenotyping [14]. Genomic DNA was extracted from each field mosquito using DNeasy Tissue Kit according to the manufacturer’s instructions.

**Table 1:** Names and genotypes (from PCR–RFLP) of samples and their sources

Countries	Specimens	PCR–RFLP genotyping	Sources
Burkina Faso	Boromo G3	*Ace.1* ^*SS*^	Larval collection
	Boromo 25	*Ace.1* ^*RS*^	Larval collection
	Dano D6	*Ace.1* ^*RS*^	Larval collection
	Boromo B2	*Ace.1* ^*RS*^	Larval collection
	Dano C7	*Ace.1* ^*RS*^	Larval collection
	Dano 34	*Ace.1* ^*RS*^	Larval collection
	Dano 33	*Ace.1* ^*RS*^	Larval collection
	Vallée du Kou A10	*Ace.1* ^*RS*^	Larval collection
	Orodara D11	*Ace.1* ^*RS*^	Larval collection
	Orodara 6.4	*Ace.1* ^*RS*^	Larval collection and crossing protocol
	Orodara 8.10	*Ace.1* ^*RS*^	Larval collection and crossing protocol
	Orodara 8.22	*Ace.1* ^*RS*^	Larval collection and crossing protocol
	Orodara A9	*Ace.1* ^*RR*^	Larval collection
	Orodara 8.13	*Ace.1* ^*RS*^	Larval collection and crossing protocol
Côte d’Ivoire	Sikensis 12	*Ace.1* ^*SS*^	Larval collection
	Daloua 11	*Ace.1* ^*SS*^	Larval collection
	Sikensis 1	*Ace.1* ^*SS*^	Larval collection
	Divo 5	*Ace.1* ^*RS*^	Larval collection
	Divo 1	*Ace.1* ^*RS*^	Larval collection
	Divo 7	*Ace.1* ^*RS*^	Larval collection
	Tiassalé 21	*Ace.1* ^*RS*^	Larval collection
	Divo 3	*Ace.1* ^*RS*^	Larval collection
	Toumodi 2	*Ace.1* ^*RS*^	Larval collection and crossing protocol
	Toumodi 8	*Ace.1* ^*RS*^	Larval collection and crossing protocol
Ghana	Okyereko	*Ace.1* ^*SS*^	Larval collection
	Cape-Coast 5	*Ace.1* ^*RS*^	Larval collection
	Koforidua 7	*Ace.1* ^*RS*^	Larval collection
	Ashaman 9	*Ace.1* ^*RR*^	Larval collection
	Ashaman 5	*Ace.1* ^*RR*^	Larval collection
	Koforidua 18	*Ace.1* ^*RR*^	Larval collection
	Ashaman 4	*Ace.1* ^*RR*^	Larval collection
	Koforidua 3	*Ace.1* ^*RR*^	Larval collection
Laboratory strain	Kisumu 1	*Ace.1* ^*SS*^	Laboratory colony
	Kisumu 8	*Ace.1* ^*SS*^	Laboratory colony
	Kisumu 3	*Ace.1* ^*SS*^	Laboratory colony
	DD1	*Ace.1* ^*RS*^	Crossing protocol
	DD2	*Ace.1* ^*RS*^	Crossing protocol
	Acerkis	*Ace.1* ^*RR*^	Laboratory colony
Togo	Baguida 77	*Ace.1* ^*RS*^	Larval collection and crossing protocol
	Baguida 99	*Ace.1* ^*RS*^	Larval collection and crossing protocol
	Baguida 6.5	*Ace.1* ^*RR*^	Larval collection and crossing protocol

### PCR–RFLP

*Ace*-*1* genotypes were first determined by using the available polymerase-chain reaction-restriction fragment length polymorphism (PCR–RFLP) analysis. The PCR primers and PCR protocol were designed according to a previously described method [16].

### TaqMan qPCR assay

TaqMan is a semi-quantitative real-time polymerase chain reaction (PCR) method that uses fluorescent probes to measure amounts of target nucleic acid. The use of two allele-specific probes carrying different fluorophores allows SNP determination in the same tube with genotype usually determined from the ratio of their intensities at the end of amplification. DNA extracts of mosquitoes of known species were genotyped individually using a standard TaqMan assay laboratory protocol [23]; run on an Agilent Stratagene MX3000 qPCR thermal cycler, and scored from bi-directional scatter plots produced by the Agilent MxPro software after amplification.

Each 10 μL PCR reaction contained 1 μL of the genomic DNA of an individual mosquito, 5 μL of SensiMixTM II Probe Kit (Bioline), 0.125 μL of Primer/Probe kit at A μM of each primer and B μM of each probe (Applied Biosystems, Foster City, CA) and 3.875 μL ddH_2_O. The PCR cycling conditions were as follows: an initial denaturation at 95 °C for 10 min, followed by 40 cycles of 95 °C for 10 s and 60 °C for 45 s. The increase in HEX and FAM fluorescence was monitored in real time by detecting fluorescence on the yellow (530 nm excitation and 555 nm emission) and green channels (470 nm excitation and 510 emission) of the qPCR thermal cycler, respectively. All samples were analysed simultaneously in the same qPCR run.

### Digital droplet PCR

Digital droplet PCR (ddPCR) combines partitioning of a qPCR reaction into many thousands of individual droplets in a water–oil emulsion, with the use of flow cytometry to count positive PCR amplicons [24]. In this work, digital droplet PCR reactions were performed using the same TaqMan primers and probes as above [23]. Reaction mixes were prepared as follows: 10 mL of 2× ddPCR Master Mix (BioRad) and 0.125 μL of Primer/Probe kit (Applied Biosystems, Foster City, CA), 2 μL of DNA template and 3.5 μL of nuclease-and protease-free water (Sigma-Aldrich Chemie Gmbh, Munich, Germany) and were added to complete a 20 μL reaction volume and mixed. The 20 μL mixture of each sample and reagents were divided into ~20,000 droplets for PCR amplification of single template molecules. Thermal cycling conditions for the assays consisted of an activation period (5 min at 95 °C) followed by 40 cycles of a two-step thermal profile comprising of a 40 cycles of a two-step thermal profile comprising of a denaturation step (30 s at 94 °C) and a combined annealing extension. The ddPCR workflow followed an established protocol [24] and data analysis was performed as described below and in Supplementary file S1 (Additional file 1). All samples were analysed simultaneously in a single ddPCR experiment.

### Quantitative real-time PCR

Primers and the protocol for a copy-number qPCR method have been described previously [17], but briefly involve amplification of three fragments of the *Ace*-*1* gene, with two endogenous reference genes used for sample normalisation, elongation factor 1-alpha (EF-1) and the P450 gene *Cyp4g16*. In *An. gambiaeCyp4g16* is located on the X chromosome allowing preliminary assessment of quantitative efficacy of amplification by comparison of males and females.

For all assays two known control samples (carrying one copy of the gene) termed calibrators (CA1 and CA2) and a no-template control (NTC) were included. The copy number of *Ace*-*1* was estimated relative to two pools of gDNA from females of two strains susceptible to organophosphates and carbamates (Kisumu and Okyereko). The reaction mixture contained 1× Power SYBR Green Master Mix (Applied Biosystems, Foster City, CA, USA), 1 pmol of each primer, 1 μL of template DNA and distilled ultra-pure water for a final reaction volume of 10 μL. The reactions were set up in a 96-well optical reaction plate (Applied Biosystems, Foster City, CA, USA) and run on an Agilent Stratagene real-time thermal cycler and analysed using Agilent’s MXPro software (Mx3005P). The PCR conditions used throughout were 10 min for 95 °C, 40 cycles of 10 s at 95 °C and 60 °C respectively, with melting curves run after each end point amplification at 1 min for 95 °C, followed by 30 s increments of 1 °C from 55 to 95 °C.

### Data analysis

TaqMan assay: raw data comprised of the final fluorescence values (dRLast), defined as the amount of fluorescence from each reporter dye at the completion of cycling, were imported into Microsoft Excel software and the ratio of dRLast FAM/dRLast HEX (‘R_TaqMan_’) was computed and used for further statistical analysis. ddPCR assay: following scanning on a QX100 droplet reader (Bio-Rad Laboratories Inc.), data were analyzed with QuantaSoft software (Bio-Rad Laboratories Inc) following published algorithms [25]. The threshold was set manually at the lowest point of the negative droplet cluster, as visualized on each of the FAM and HEX probes. The ddPCR results were expressed as the number of droplets where amplification has or has not occurred (positive and negatives, respectively). In the case of this study, where a FAM/HEX duplex assay was performed, four discrete clusters of droplets are possible: (1) no target allele (negative FAM/negative HEX), indicative of a negative control or failed assay; (2, 3) only one of the targets is positive (negative FAM/positive HEX, or positive FAM/negative HEX), indicative of a homozygote; or (4) both targets are positive (positive FAM/positive HEX), indicative of a heterozygote. Results from (2), (3) and (4) can be used to compute the average number of copies of PCR amplicons for each allele (λ_*Ace*-*1*_^*R*^ and λ _*Ace*-*1*_^*S*^, respectively for resistant and susceptible alleles at the *Ace*-*1* locus) based on the fraction of positive droplets and Poisson modeling using the following formula {a}: −ln(1−(p/T)) where p is the number of positive droplets containing each amplified allele, and T is the number of positives droplets. From the data the number of copies from the ratio of λ estimates for each allele was determine.

For the ddPCR assay the ratio data was first transformed to produce a distribution close to normal (see Additional file 1), using the logarithm of R_ddPCR_, given by the equation {b}: X = ln(R_ddPCR_). To test the difference in the logarithm of the observed ratio between *Ace*-*1*^*R*^ and the reference (*Ace*-*1*^*S*^) from zero, the standard deviation of X (estimated from the equation {b} above) was calculated. The variance of the log ratio X was determined using the equation {c}: $\sigma^{ 2} {\text{X}} = \left( { 1 - {\text{EXP}}\left( {\lambda 1 1 9 {\text{S}}} \right)} \right)/({\text{T}} \times \lambda^{ 2} 1 1 9 {\text{S}} \times {\text{EXP(}}\lambda 1 1 9 {\text{S}})) + \left( { 1 - {\text{EXP}}\left( {\lambda 1 1 9 {\text{G}}} \right)} \right)/\left( {{\text{C}} \times \lambda^{ 2} 1 1 9 {\text{S}} \times {\text{EXP}}\left( {\lambda 1 1 9 {\text{G}}} \right)} \right)$. With the variance in the log ratio, the upper and lower 95 % CI were calculated (see Additional file 1) for others formulas used in this section. Finally, the X values and their standard deviations for each test sample were plotted.

Copy-number qRT-PCR: data analysis followed the delta–delta Ct (∆∆Ct) method of relative quantification [26] to estimate copy numbers of the *Ace*-*1* gene (averaged across the three primer pairs) as described in Edi et al. [17]. To group heterozygous individuals recorded from the PCR–RFLP analysis (Table 1) into different assumptive genotypes K-means clustering (using squared Eucliden distances and an iterative method) was applied on the Z-scores of the correspondent data obtained from both TaqMan and ddPCR methods. The variances in their scores explained by the clustering solution were calculated using analysis of variance (ANOVA). The statistical software package *ade4* in R-project version 3.1.2 [27] was used to perform these analysis.

## Results

Using PCR–RFLP assays, a total of 41 female *An. gambiae* and *An. coluzzii* mosquitoes collected from 14 locations across four countries were analysed. These comprised of six specimens of known PCR–RFLP *Ace*-*1* genotype (three homozygous *Ace*-*1*^*SS*^; two heterozygous *Ace*-*1*^*RS*^ and one homozygous *Ace*-*1*^*RR*^); and 35 individuals of unknown *Ace*-*1* genotype. Of these 35 field-collected specimens 23 (65.7 %) typed as heterozygous, seven (20 %) were homozygous *Ace*-*1*^*RR*^ and five (14.3 %) were homozygous *Ace*-*1*^*SS*^ (Table 1).

**Fig. 1:**
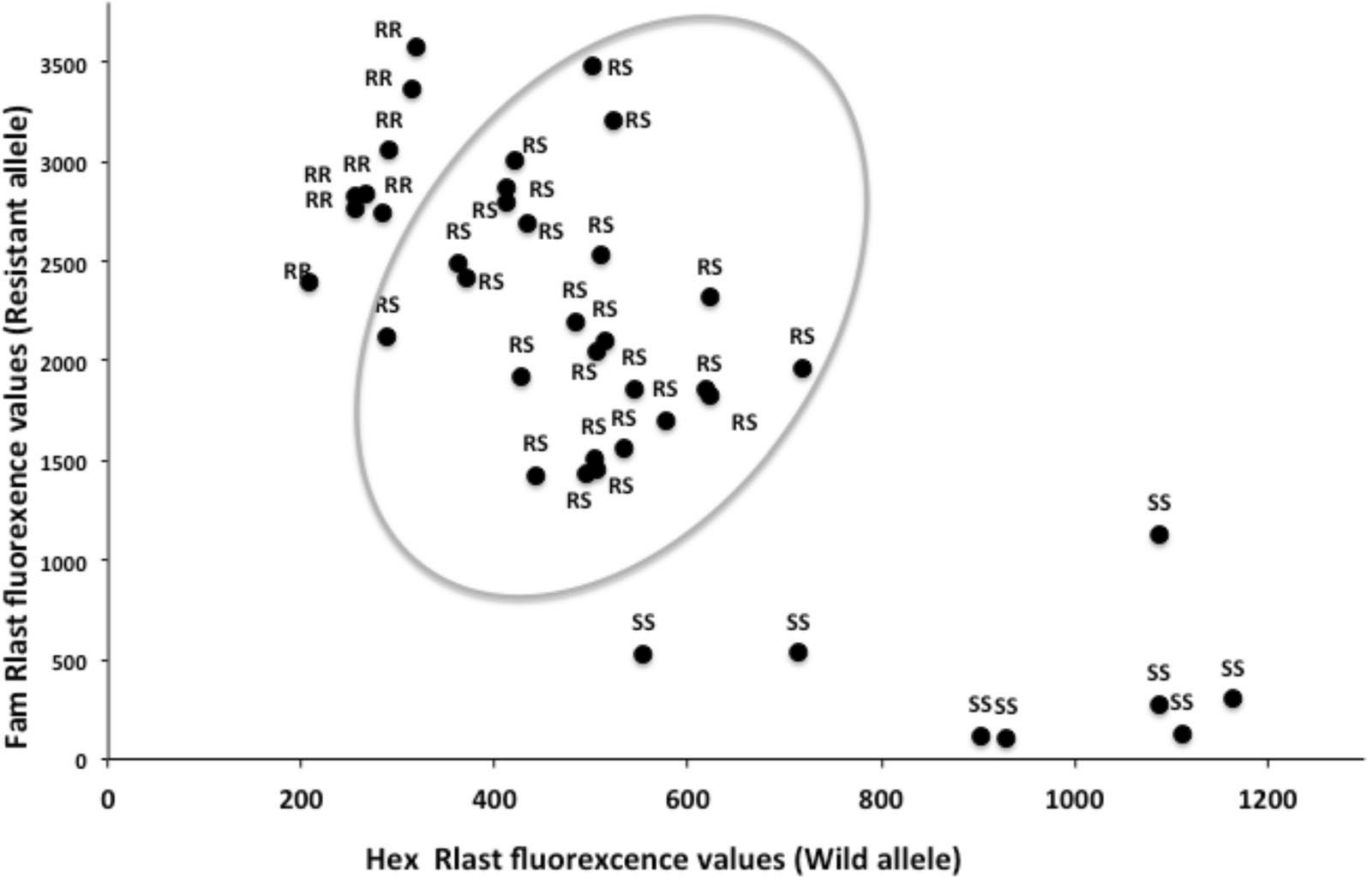
Scatter plot analysis of TaqMan fluorescence data. dRlast fluorescence values of the FAM labelled probe (specific for the *Ace*-*1* ^*R*^ mutation) are plotted against the HEX labelled probe (specific for the wild type *Ace*-*1* ^*S*^ allele). RR, genotype *Ace*-*1* ^*RR*^; RS, genotype *Ace*-*1* ^*RS*^; SS, genotype *Ace*-*1* ^*SS*^. The *circle* indicates the limit of heterozygous specimens

The same 41 DNA samples were genotyped using the TaqMan assay, plotting baseline-corrected endpoint values (dRLast) for each dye (FAM and HEX) in a bi-directional scatter-plot (Fig. 1). Classically, high HEX fluorescence alone indicates a homozygote for the wild allele of acetylcholinesterase enzyme termed *Ace*-*1*^*SS*^ (homozygote susceptible), high FAM fluorescence alone indicates a homozygote resistant (*Ace*-*1*^*RR*^) and high signals in each dye indicate a heterozygote termed *Ace*-*1*^*RS*^. The diffuse and fragmented nature of apparent heterozygotes (Fig. 1) means that simple genotypic designation cannot be easily made using the standard method of calling [23].

After droplet digital PCR reactions, the average number of accepted droplet reactions in each ddPCR was 12365 ± 1876. By considering the *Ace*-*1*^*S*^ allele as the reference the R-value that is the ratio of λ (number of copies of PCR amplicons for each allele) estimates for each allele (R_ddPCR_ = λ_*Ace*-*1*_^*R*^/λ_*Ace*-*1*_^*S*^) was determined (see Additional file 1).

**Fig. 2:**
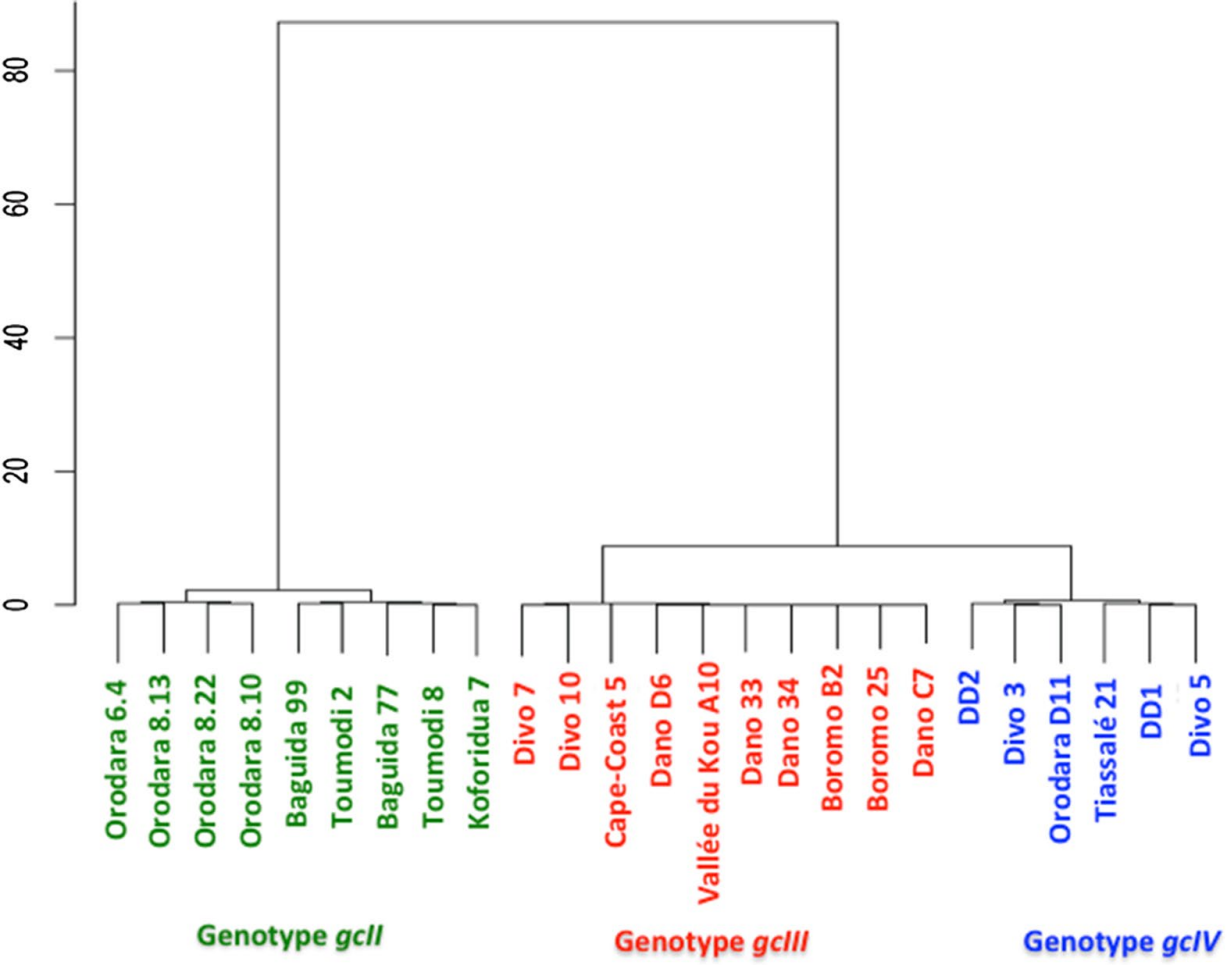
Dendrogram from *k*-means clustering analysis showing genotype calling groups obtained from specimens bearing both *Ace*-*1* alleles

**Fig. 3:**
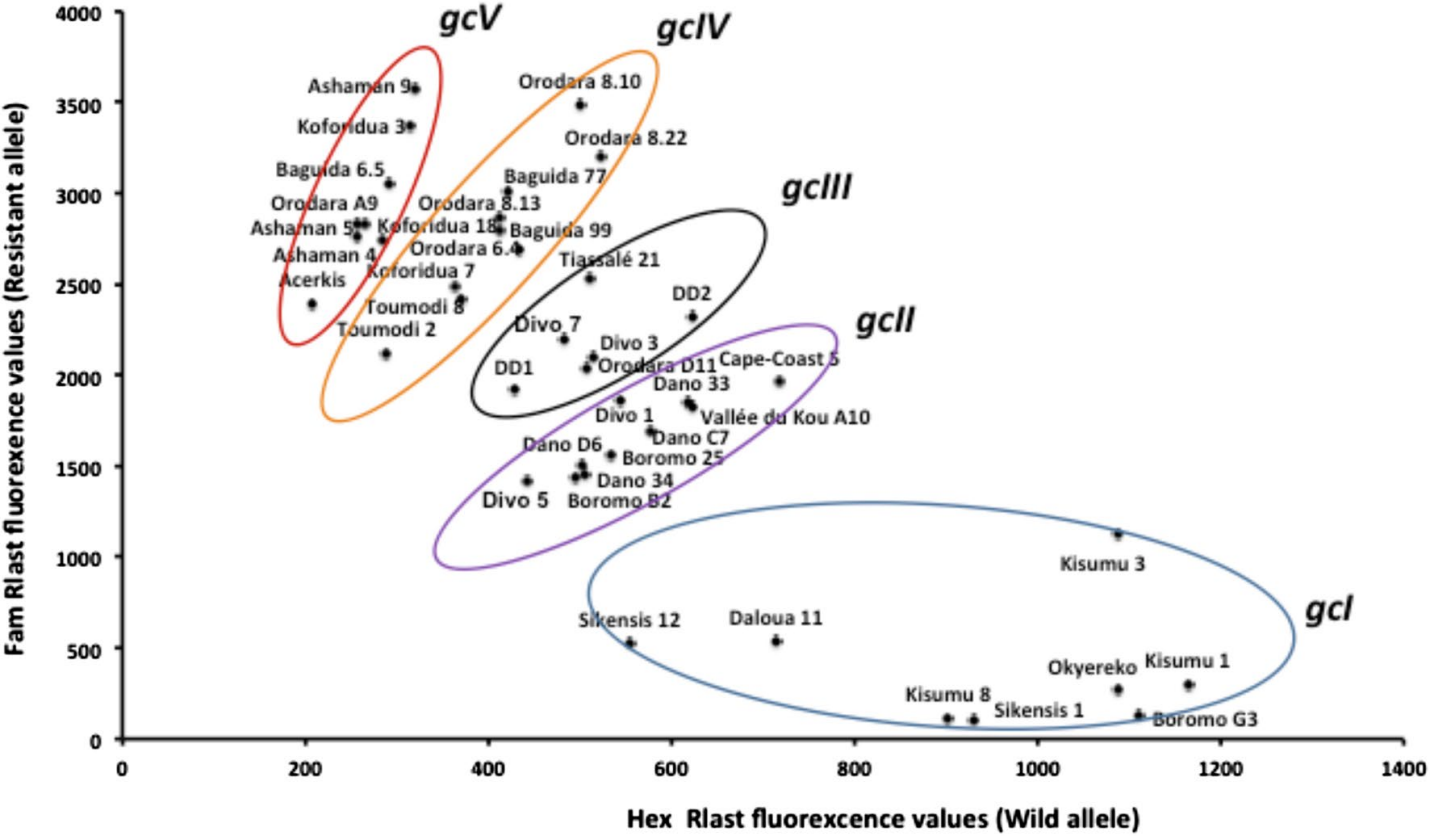
Scatter plot analysis of TaqMan fluorescence data showing different genotype clusters, denoted by *different* *coloured circles*

**Fig. 4:**
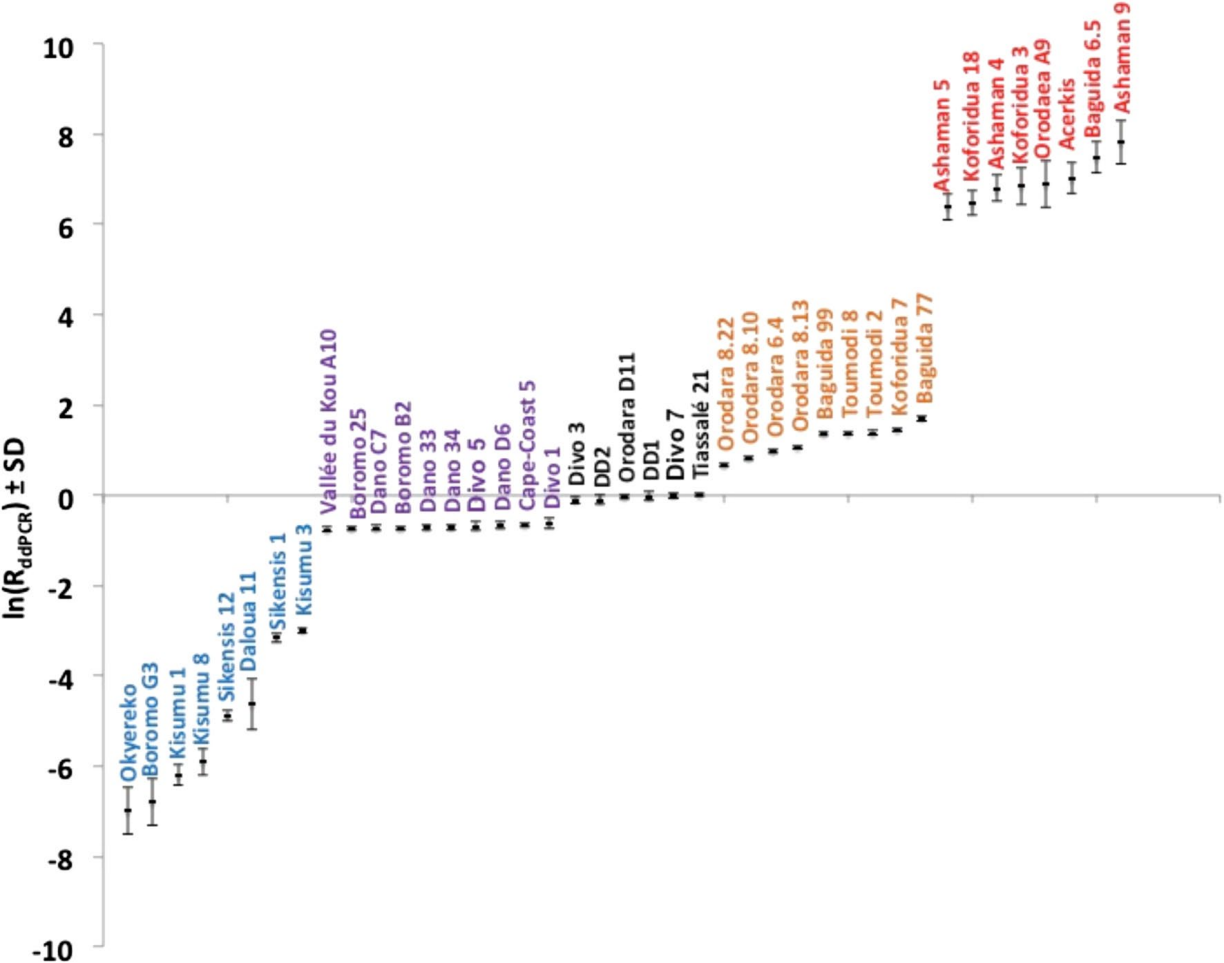
Plot of the log (*R* = *λ*_119S_/*λ*_119G_) computed from the raw data obtained from ddPCR assays

K-means cluster analysis using separately TaqMan (R_TaqMan_) and ddPCR (R_ddPCR_) data was used to group individuals typing as heterozygotes in the PCR–RFLP assay (Fig. 1). Samples were clustered into three genotype groups (named *gcII*, *gcIII* and *gcIV*) as shown in Fig. 2. Based on these clusters, manual annotation to the TaqMan scatter plot highlighted five clusters: the three recorded above and homozygous susceptible individuals termed *gcI* (genotype cluster I) and homozygous resistant individuals termed *gcV* (genotype cluster V) (Fig. 3). Results from the ddPCR assay are shown in Fig. 4 with samples arranged left to right in order of increasing ratio of resistance allele signal (λ_*Ace*-*1*_^*R*^/λ_*Ace*-*1*_^*S*^) values; the five groups of specimen genotypes are indicated.

**Fig. 5:**
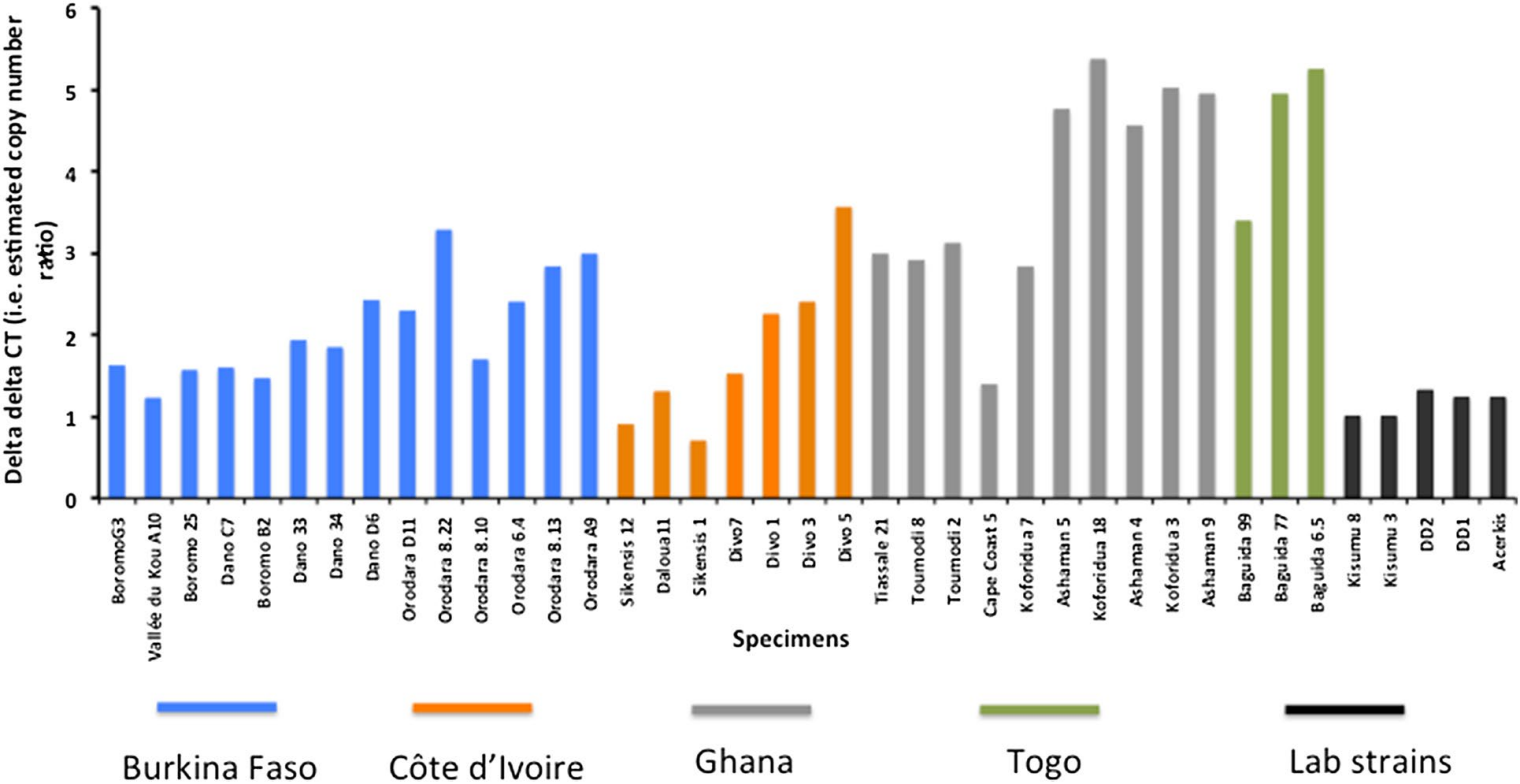
*Ace*-*1* gene copy number level estimated by qRT-PCR

**Fig. 6:**
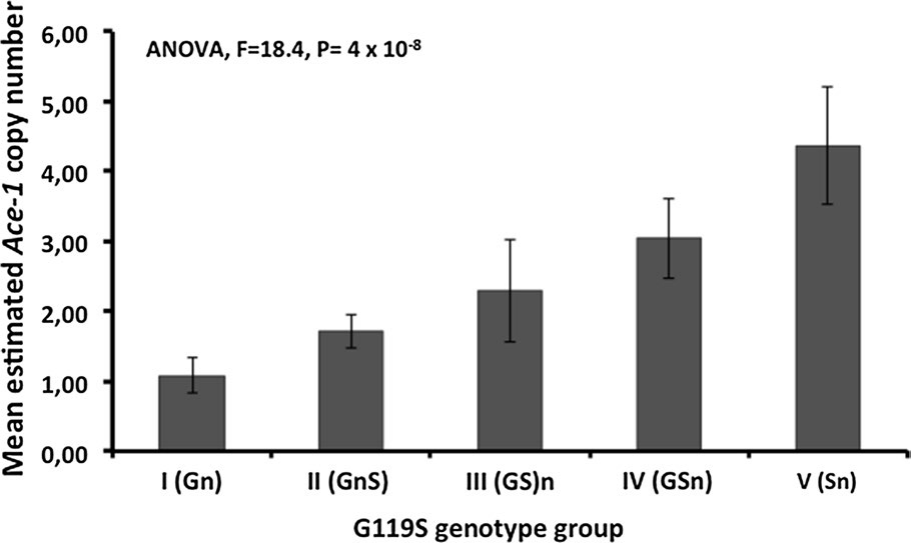
Mean *Ace*-*1* gene copy number level (with standard deviations) estimated by qRT-PCR for each of the genotype groups identified by TaqMan and ddPCR

*Ace*-*1* gene copy number varied dramatically among the samples assayed. Assuming that the calibrators used in this study have only two copies of the *Ace*-*1* gene as expected for a diploid with a single copy gene, some samples carry an estimated five copies of the *Ace*-*1* locus. Results indicate both a high rate of CNVs at this locus and a broad geographical spread (Fig. 5). There was a strong and highly significant difference in copy number (estimated from the qRTPCR) among the groups identified by TaqMan and ddPCR (ANOVA: F_4,38_ = 18.4, P = 4 × 10^−8^) with a progressive increase from a single-copy average for susceptible homozygotes (Gn), suggesting that all or most are unduplicated, to resistant homozygotes (Sn) which averaged in excess of four gene copies (Fig. 6). This suggests that resistant alleles are far more likely to be duplicated than susceptible and, importantly, that groupings from TaqMan (or ddPCR) give a meaningful semi-quantitative indication of copy number variation detected by the quantitative, but not allele-specific, qRTPCR method.

## Discussion

Occurrence of duplication of *Ace*-*1* has been detected in both *Culexquinquefasciatus* and *An. gambiae* [14, 28] but the lack of a specific test (enzymatic or molecular) to detect duplications has been an impediment to understanding their nature and impacts. Identification of individuals harboring duplicated alleles by designing crosses and observing progeny survival in bioassays [14] is too time-consuming to allow proper interpretation of CNV in *Ace*-*1*^*RS*^ genotypes scored using the traditional PCR–RFLP technique [29]. Indeed these represent a highly heterogeneous group, which in *An. coluzzii* from Tiassale (which almost all type as heterozygotes in PCR–RFLP), exhibit significantly variable bioassay survival [17]. With *Ace*-*1* CNV apparently now spread across such a broad area of West Africa, assays giving insight into CNV variation of the kind which were evaluate here, are urgently required.

In addition to PCR–RFLP, three different molecular techniques were applied on the same mosquito specimens (either collected from field or provided from laboratory strains). TaqMan genotyping is a high throughput and highly accurate methodology widely used for detection of target site mutations in mosquitoes [30, 31]. ddPCR has been adopted for a number of applications, including studies of copy number variation involving allelic discrimination or imbalance, single cell gene expression, detection of low copy number nucleic acid targets [32, 33] and of point mutations. Though giving high precision estimation of allelic balance for heterozygotes, ddPCR cannot give quantitative insight into CNV level in homozygotes or GnSn heterozygotes with an equal allele balance. With the TaqMan method, instead of having clearly distinct expected cluster patterns [23] it was displayed a large spectral distribution of heterozygous individuals that rendered manual genotype calling of subgroups difficult. Therefore attempts have been made to automatically assign genotype using K-means on the transformed fluorescence data generated by both TaqMan and ddPCR techniques. A clear separation of heterozygous genotype subgroups was obtained, to supplement the straightforward identification of the distinct homozygous groups, and these clusters exhibited significant variation in mean CNV level assessed by the copy number detection qRTPCR (Fig. 4). This cross-comparability between techniques suggests that where separation of heterozygote sub-groups is the primary aim, TaqMan and ddPCR assays can provide useful semi-quantitative estimation of copy number variation. Most, if not all, of the susceptible homozygote individuals in this study appear to possess only a single copy of *Ace*-*1*, consistent with results from Accra, Ghana [34], therefore application of the qRTPCR assay to samples genotyping as glycine homozygotes may be a lesser priority. However to investigate the resistance consequences of variation in resistant allele copy number in resistant (serine) homozygotes, genotyping will need to be supplemented by SYBR green qPCR.

By interpreting each genotype cluster position in the Fig. 3 following genotype calls are suggest (see Table 2):

**Table 2:** Different clusters observed, with suggested genotype calls obtained from both TaqMan and ddPCR data followed by K-means clustering analysis

N°	Genotype cluster (*gc*)	Samples	Estimates genotypes
1	I	Okyereko	Gn: Homozygous susceptible individuals (*Ace*-*1* ^*nSS*^) with *Ace*-*1* gene copy number greater than or equal to 1
2		Boromo G3	
3		Kisumu 1	
4		Kisumu 8	
5		Sikensis 12	
6		Daloua 11	
7		Sikensis 1	
8		Kisumu 3	
9	II	Vallée du Kou	GnS: Heterozygous individuals (*Ace*-*1* ^*GnS*^) with more susceptible copies than resistant copies
10		Boromo 25	
11		Dano C7	
12		Boromo B2	
13		Dano 33	
14		Dano 34	
15		Divo 5	
16		Dano D6	
17		Cape-Coast 5	
18		Divo 1	
19	III	Divo 3	(GS)n: Heterozygous individuals (*Ace*-*1* ^(*GS*)*n*^) with equal of susceptible and resistant allele and Ace-1 gene copy number greater than or equal to 1
20		DD2	
21		Orodara D11	
22		DD1	
23		Divo 7	
24		Tiassalé 21	
25	IV	Orodara 8.22	GSn: Heterozygous individuals (*Ace*-*1* ^*GSn*^) with more resistant allele than susceptible one
26		Orodara 8.10	
27		Orodara 6.4	
28		Orodara 8.13	
29		Baguida 99	
30		Toumodi 8	
31		Toumodi 2	
32		Koforidua 7	
33		Baguida 77	
34	V	Ashaman 5	Sn: homozygous resistant individuals (*Ace*-*1* ^*Sn*^) with *Ace*-*1* gene copy number greater than or equal to 1
35		Koforidua 18	
36		Ashaman 4	
37		Koforidua 3	
38		Orodaea A9	
39		Acerkis	
40		Baguida 6.5	
41		Ashaman 9

- Gn: genotype cluster I = homozygous susceptible individuals (*Ace*-*1*^*nSS*^).
- GnS: genotype cluster II = heterozygous individuals (*Ace*-*1*^*GnS*^).
- (GS)n: genotype cluster III = heterozygous individuals (*Ace*-*1*^(*GS*)*n*^).
- GSn: genotype cluster IV = heterozygous individuals (*Ace*-*1*^*GSn*^).
- Sn: genotype cluster V = homozygous resistant individuals (*Ace*-*1*^*Sn*^).

Previous studies discovered, what appeared to be rare, duplications of *Ace*-*1* in *An. gambiae* (and *An. coluzzii*) [11, 28] via the occurrence of a resistant and two distinct susceptible alleles in sequence data from single individuals. In contrast, results of the present study indicated that duplication event is very prevalent and spans a range of possible genotypes involving multiple resistant alleles. Furthermore, the highest copy number was recorded in individuals with a strong imbalance of resistant to susceptible copies in contrast with previous findings in *Culex* [14]. *Anopheles gambiae* thus seems to exhibit far greater complexity of duplication at the *Ace*-*1* gene.

## Additional file

10.1186/s12936-015-1026-3: Statistical analysis of row data obtained from ddPCR method. The data provided represent the average number of copies of PCR amplicons and the number of copies from the ratio of λ estimates for each allele in each sample.
